# *Lentinula edodes* substrate formulation using multilayer perceptron-genetic algorithm: a critical production checkpoint

**DOI:** 10.3389/fmicb.2024.1366264

**Published:** 2024-05-21

**Authors:** Naser Safaie, Mina Salehi, Siamak Farhadi, Ali Aligholizadeh, Valiollah Mahdizadeh

**Affiliations:** ^1^Department of Plant Pathology, Faculty of Agriculture, Tarbiat Modares University, Tehran, Iran; ^2^Department of Plant Genetics and Breeding, Faculty of Agriculture, Tarbiat Modares University, Tehran, Iran; ^3^Seed and Plant Improvement Institute, Agricultural Research, Education and Extension Organization (AREEO), Karaj, Iran

**Keywords:** Shiitake, medicinal and edible mushroom, running, optimization, artificial neural network

## Abstract

Shiitake (*Lentinula edodes*) is one of the most widely grown and consumed mushroom species worldwide. They are a potential source of food and medicine because they are rich in nutrients and contain various minerals, vitamins, essential macro- and micronutrients, and bioactive compounds. The reuse of agricultural and industrial residues is crucial from an ecological and economic perspective. In this study, the running length (RL) of *L. edodes* cultured on 64 substrate compositions obtained from different ratios of bagasse (B), wheat bran (WB), and beech sawdust (BS) was recorded at intervals of 5 days after cultivation until the 40^th^ day. Multilayer perceptron-genetic algorithm (MLP-GA), multiple linear regression, stepwise regression, principal component regression, ordinary least squares regression, and partial least squares regression were used to predict and optimize the RL and running rate (RR) of *L. edodes*. The statistical values showed higher prediction accuracies of the MLP-GA models (92% and 97%, respectively) compared with those of the regression models (52% and 71%, respectively) for RL and RR. The high degree of fit between the forecasted and actual values of the RL and RR of *L. edodes* confirmed the superior performance of the developed MLP-GA models. An optimization analysis on the established MLP-GA models showed that a substrate containing 15.1% B, 45.1% WB, and 10.16% BS and a running time of 28 days and 10 h could result in the maximum *L. edodes* RL (10.69 cm). Moreover, the highest RR of *L. edodes* (0.44 cm d^−1^) could be obtained by a substrate containing 30.7% B, 90.4% WB, and 0.0% BS. MLP-GA was observed to be an effective method for predicting and consequently selecting the best substrate composition for the maximal RL and RR of *L. edodes*.

## 1 Introduction

The cultivation of the mushrooms not only lessens the environmental effects of the wastes used as substrate but also provides an economically viable alternative for producing high-quality and beneficial food and precious metabolites (Israilides and Philippoussis, 2003). Shiitake, *Lentinula edodes* (Berk.) Pegler, has been cultivated for thousands of years. *L. edodes* is one of the best-known medicinal and edible mushrooms worldwide, especially in East Asia, due to its widespread use in food and traditional medicines (Xu et al., 2014; Mahdizadeh et al., 2021; Ahmad et al., 2023). This valuable medicinal mushroom is used to treat influenza, tumors, high blood pressure, heart disease, obesity, age-related sexual dysfunction, liver problems, diabetes, fatigue, weakness, high cholesterol, and respiratory diseases (Bisen et al., 2010; Ahmad et al., 2023). Shiitake is nourishing and appetizing as a food and exhibit several pharmacological properties, including antiviral, immunomodulatory, antifungal, antibacterial, antiviral and anticancer effects (Bisen et al., 2010; Diallo et al., 2020; García-Cruz et al., 2020; Ahmad et al., 2023). The lignan-rich fraction extracted from *L. edodes* mycelial culture medium is promising for the treatment of AIDS and hepatitis B (Bisen et al., 2010).

The preferred substrate for *L. edodes* is hardwood sawdust supplemented with rice bran, wheat bran and millet powder (Philippoussis et al., 2007). Bagasse substrate supplemented with rice bran improved mushroom quality and biological efficiency of shiitake (Rossi et al., 2003). Bagasse is an important solid residue in the sugarcane industry, and hundreds of millions of tons of bagasse are produced worldwide every year (Mahmud and Anannya, 2021). The use of bagasse (Selman-Housein et al., 2000) and other agricultural residues (Devi et al., 2017), including wheat bran, is crucial from both environmental and economic perspectives. Beech sawdust is obtained in abundance from the timber industry (Jahedi et al., 2023). The reuse of these solid wastes is also crucial for environmental reasons (Gordić et al., 2014; Jahedi et al., 2023). In this study, different ratios of bagasse (B), beech sawdust (BS), and wheat bran (WB) were used as growing substrates to determine the compatibility of agricultural and industrial residues for shiitake production. The ratio of substrate components should be optimized to achieve the best running of *L. edodes*. A detailed analysis of the effects of the ratio of growing substrate components on the running length (RL) and running rate (RR) of *L. edodes* and their optimal quantity selection would lay the foundation for the commercialization of the conversion of agro-industrial residues into foods with therapeutic active ingredients. However, optimizing the ratio of growing substrate components to achieve the maximum running is expensive and time-consuming. Evaluating the relationship between the input variables “ratios of substrate components (B, BS, and WB) and running time” and the output variables “RL and RR of *L. edodes*” could simplify the optimization of the growing substrate for production of this valuable medicinal and edible mushroom. Analyses of biological data have been conducted using multivariate statistical methods, including multiple linear regression (MLR), stepwise regression (SR), principal component regression (PCR), ordinary least squares regression (OLSR), and partial least squares regression (PLSR; Wold, 1966; Farhadi et al., 2020; Salehi et al., 2020b). Growth is a highly nonlinear and complex biological process involving multiple interconnected signaling and biochemical pathways (Olas et al., 2020). The optimal conditions for a complex process can be accurately predicted using efficient nonlinear computational algorithms (Struik et al., 2005; Gallego et al., 2011). Traditional prediction and modeling methods, including regression models, have negligible nonlinear fitting and prediction capabilities (Farhadi et al., 2020; Salehi et al., 2020a,b). Artificial intelligence (AI) is used to address issues that cannot be explained using traditional computing techniques. One of the key aspects of AI is artificial neural networks (ANNs), which discover multifactorial nonlinear relationships between the output and input data (Farhadi et al., 2020; Hesami et al., 2020; Hesami and Jones, 2020; Salehi et al., 2020a; Jahedi et al., 2023). ANNs are brain-inspired systems that mimic the human brain’s ability to sense and think in a simplified manner to process information and recognize patterns in systems that involve ambiguity and uncertainty (Patnaik, 1999; Agatonovic-Kustrin and Beresford, 2000). ANNs learn through experience and gain intelligence by discovering hidden patterns and relationships (Agatonovic-Kustrin and Beresford, 2000). Response surface methodology (Kovalchuk et al., 2017), multivariate adaptive regression splines (Akin et al., 2020), and multilayer perceptron-genetic algorithm (MLP-GA; Jahedi et al., 2023) have been used for culture media optimization in plant and fungal research. Multilayer perceptron (MLP) is an effective tool in solving complicated non-linear problems, handles the massive data set well, quickly predicts after training, and obtains the same accuracy even with small data. Disadvantages of MLP include that the degree to which the dependent variable influences each independent variable is unknown, some hyperparameters of the MLP, such as the number of hidden neurons and layers, must be tuned, which requires time and power, model performance depends on the training quality, and the MLP is sensitive to feature scaling (Pedregosa et al., 2011). MLP, one of the most well-known feedforward neural networks, exhibits superior prediction accuracy compared to traditional statistical methods for calculating mathematical functions for analyzing and interpreting various unpredictable data sets (Salehi et al., 2020a; Jahedi et al., 2023). However, there are numerous problems in designing and training ANNs. Due to a large number of hidden neurons, the training time increases and data overfitting occurs. Low accuracy rates are caused by a few hidden neurons (Matignon, 2005). In addition, one of the main problems is the allocation of weights in the MLP structure which directly affects the model performance. Network topology and learning algorithm parameters control the weights. Learning rates, the number of memory taps, and the number of hidden nodes and layers are the network factors that can influence ANN performance (Tahmasebi and Hezarkhani, 2009). To address these issues, ANNs have been combined with other optimization techniques, such as genetic algorithms (GA; Plumb et al., 2005; Salehi et al., 2020a).

GA, developed by Holland (1992), is a popular evolutionary algorithm that excels in finding answers to problems and has been used to optimize bioprocesses (Osama et al., 2015; Farhadi et al., 2020; Salehi et al., 2020a; Jahedi et al., 2023). The GA is a search algorithm inspired by genetics and natural selection (Holland, 1992). GAs apply biologically inspired operators, including crossover, mutation, and selection, to obtain high-quality optimization solutions (Katoch et al., 2021). The principles of GA are the creation of the initial population of search solutions (chromosomes), followed by selecting the superior search solutions for crossover using a roulette wheel selection method and determining the best solution (optimal value, fittest chromosome) among them (Holland, 1992). GA obtains solutions improving over time, requires no derivative information, displays superior parallel capabilities, optimizes continuous functions and discrete functions, and multi-objective problems, is the best option for a wide range of optimization problems, and can handle a broad search space. However, GA is not an efficient method for solving simple problems and ones with available derivative information. Additionally, GAs may be computationally costly, especially when working with large problem spaces or complex fitness evaluations. Also, the quality of the final solution found using GA to a problem is not guaranteed (Sivanandam and Deepa, 2008). This study attempted to automatically determine the optimal number of neurons using GA to improve the prediction accuracy and determine the optimized values of the inputs “(B, BS, and WB ratio)” for the maximum mycelial RL and RR of shiitake (*L. edodes*). Furthermore, the performances of the regression and MLP-GA models were evaluated in terms of the prediction accuracy of the output variables optimized for the maximum mycelial RL and RR of *L. edodes*, and the most important inputs (factors) were detected for the highest RL and RR of shiitake.

## 2 Materials and methods

Shiitake (*L. edodes*) strain VM230 was obtained from the culture collection of the Plant Pathology Department at Tarbiat Modares University (Mahdizadeh et al., 2021).

### 2.1 Preparation of media

Bagasse (B), beech sawdust (BS) and wheat bran (WB) were used as components for the growing substrate. First, they were pulverized with a blender and passed through a sieve (pore size of 5 mm in diameter; Supplementary Figure S1). Different ratios of substrate components (Table 1) were mixed manually. It is noteworthy that the ratios reported for each component (B, WB, and BS; Table 1) are percentages of 10 g of each component. Each substrate was poured halfway (1.4 mL) into a glass Pasteur pipette (0.6 mm diameter, 11.5 cm height, and 2.8 mL volume). The Pasteur pipettes were then placed horizontally so that the substrate was placed on one side of the glass pipette and the other side was left empty for ventilation. The Pasteur pipettes were placed in a tray and some distilled water was added. They were then kept in the dark at 25°C for 12 h so that the substrate in the Pasteur pipettes completely absorbed the water. A sample of the substrates was weighed, gently dried in an oven at 45°C, and then weighed again to determine the final water content (55%). The substrates were then autoclaved twice at 121°C and 1.5 atm for 40 min at an interval of 24 h. One mycelial agar plug (4 mm diameter) per replicate was cut from the edge of the growing shiitake (*L. edodes*) colony previously cultured on the culture medium prepared from the extract “B (25%) + WB (50%) + BS (25%),” using a sterilized cork-borer, cultured in the Pasteur pipette (Supplementary Figure S2) containing the different ratios of substrate components listed in Table 1 and kept in the dark at 25°C. The RL (cm) of *L. edodes* cultured on each substrate was recorded at 5-day intervals after cultivation until the day 40. The running rate (RR; cm d^−1^) of *L. edodes* was calculated using Eq. (1).


(1) $$\text{Running}\;\text{rate}\;\left(\text{cm}\;day^{-1}\right)=\frac{\text{Running}\;\text{length}\;\left(\text{cm}\right)}{\text{Running}\;\text{time}\;\left(\text{day}\right)}$$

**Table 1:** Different substrate compositions for the cultivation of shiitake (*Lentinula edodes*) and the day (after cultivation) on which the recorded highest statistically significant running length (RL).

	Bagasse (%)	Wheat bran (%)	Beech sawdust (%)	Substrate name	Days-to-highest RL
S_1_	0	0	0	S^0/0/0^	-
S_2_	0	0	25	S^0/0/25^	40
S_3_	0	0	50	S^0/0/50^	40
S_4_	0	0	100	S^0/0/100^	40
S_5_	0	25	0	S^0/25/0^	30
S_6_	0	25	25	S^0/25/25^	30
S_7_	0	25	50	S^0/25/50^	30
S_8_	0	25	100	S^0/25/100^	30
S_9_	0	50	0	S^0/50/0^	30
S_10_	0	50	25	S^0/50/25^	30
S_11_	0	50	50	S^0/50/50^	30
S_12_	0	50	100	S^0/50/100^	30
S_13_	0	100	0	S^0/100/0^	30
S_14_	0	100	25	S^0/100/25^	30
S_15_	0	100	50	S^0/100/50^	30
S_16_	0	100	100	S^0/100/100^	30
S_17_	25	0	0	S^25/0/0^	35
S_18_	25	0	25	S^25/0/25^	35
S_19_	25	0	50	S^25/0/50^	35
S_20_	25	0	100	S^25/0/100^	30
S_21_	25	25	0	S^25/25/0^	30
S_22_	25	25	25	S^25/25/25^	30
S_23_	25	25	50	S^25/25/50^	30
S_24_	25	25	100	S^25/25/100^	30
S_25_	25	50	0	S^25/50/0^	25
S_26_	25	50	25	S^25/50/25^	30
S_27_	25	50	50	S^25/50/50^	30
S_28_	25	50	100	S^25/50/100^	30
S_29_	25	100	0	S^25/100/0^	25
S_30_	25	100	25	S^25/100/25^	25
S_31_	25	100	50	S^25/100/50^	25
S_32_	25	100	100	S^25/100/100^	25
S_33_	50	0	0	S^50/0/0^	35
S_34_	50	0	25	S^50/0/25^	35
S_35_	50	0	50	S^50/0/50^	30
S_36_	50	0	100	S^50/0/100^	30
S_37_	50	25	0	S^50/25/0^	30
S_38_	50	25	25	S^50/25/25^	30
S_39_	50	25	50	S^50/25/50^	30
S_40_	50	25	100	S^50/25/100^	30
S_41_	50	50	0	S^50/50/0^	30
S_42_	50	50	25	S^50/50/25^	25
S_43_	50	50	50	S^50/50/50^	25
S_44_	50	50	100	S^50/50/100^	25
S_45_	50	100	0	S^50/100/0^	25
S_46_	50	100	25	S^50/100/25^	25
S_47_	50	100	50	S^50/100/50^	30
S_48_	50	100	100	S^50/100/100^	30
S_49_	100	0	0	S^100/0/0^	40
S_50_	100	0	25	S^100/0/25^	40
S_51_	100	0	50	S^100/0/50^	40
S_52_	100	0	100	S^100/0/100^	40
S_53_	100	25	0	S^100/25/0^	30
S_54_	100	25	25	S^100/25/25^	30
S_55_	100	25	50	S^100/25/50^	30
S_56_	100	25	100	S^100/25/100^	30
S_57_	100	50	0	S^100/50/0^	35
S_58_	100	50	25	S^100/50/25^	30
S_59_	100	50	50	S^100/50/50^	30
S_60_	100	50	100	S^100/50/100^	30
S_61_	100	100	0	S^100/100/0^	30
S_62_	100	100	25	S^100/100/25^	25
S_63_	100	100	50	S^100/100/50^	25
S_64_	100	100	100	S^100/100/100^	25

### 2.2 Experimental design

The experiment was conducted based on a randomized complete block design (RCBD) with factorial arrangement containing four factors: the bagasse ratio with four levels, i.e., 25% (2.5 g), 50% (5 g), 75% (7.5), and 100% (10 g), the wheat bran ratio with four levels (25%, 50%, 75%, and 100%), the beech sawdust ratio with four levels (25%, 50%, 75%, and 100%), and the running time with eight levels (days 5, 10, 15, 20, 25, 30, 35, and 40 after cultivation), given 512 treatments, and four replicates. The substrate compositions was listed in Table 1.

### 2.3 Statistical analysis

Parametric statistics were used because the normality hypothesis and homogeneity of variance were satisfied. Analysis of variance (ANOVA) was used to assess the factorial experiment based on the randomized complete block design (RCBD). Least significant difference was used to compare the growing substrate compositions (64 substrate compositions are presented in Table 1) in a time-dependent manner to identify the highest significant RL of *L. edodes* during the running time for each substrate. Mean comparisons were performed using Student–Newman–Keuls for the factorial experiments (examining the effects of different ratios of substrate components on the RL and RR of *L. edodes* in B-ratio-, WB-ratio-, and BS-ratio-dependent manners).

### 2.4 Model development

Data were normalized using the Box–Cox transformation (Box and Cox, 1964) before the machine learning algorithm was tested. No outliers were identified by the principal component analysis. The performance of each tested model on the dataset (2,048 and 256 data lines for RL and RR, respectively) was calculated using an eight-fold cross-validation method with 10 repeats, and the model with the highest prediction accuracy for unknown data from the dataset was determined.

#### 2.4.1 Multilayer perceptron model

Three-layered MLP modeling was used to determine the effects of the ratio of substrate components, namely B, BS, and WB, and running time on the RL and RR of *L. edodes*.

**Figure 1:**
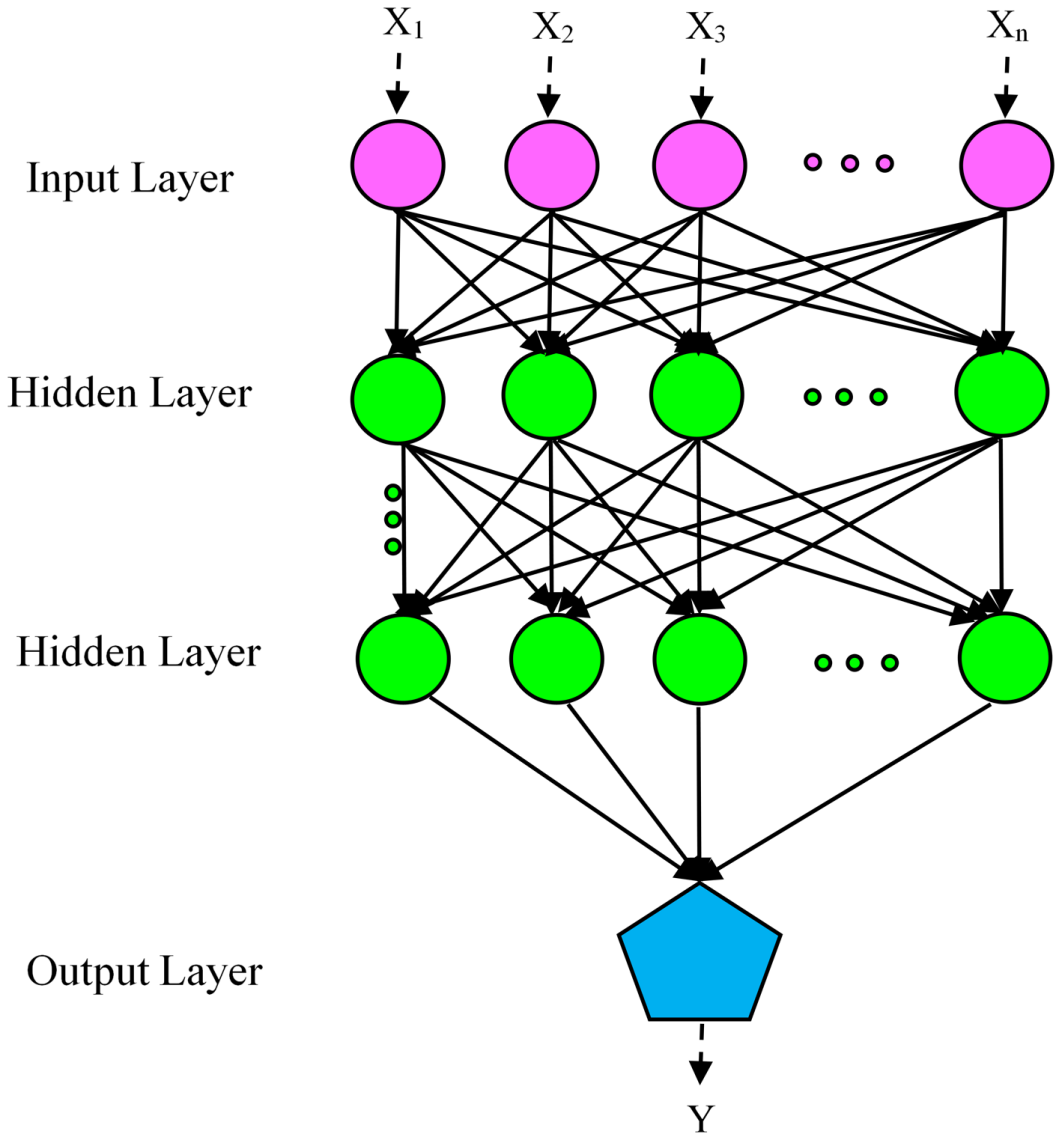
Schematic of multilayer perceptron (MLP) architecture.

MLP, a supplement to the feedforward neural network, consists of three distinct layers: input, hidden, and output layers, as shown in Figure 1. The input layer (visible layer) feeds the input variables into the second layer (hidden layer). Between input and output lie one or more intermediate layers, so-called hidden layers, which form the internal brain of the network. The output layer predicts the output based on the information transmitted by the input layer. The predicted output is compared with the actual output and the error is calculated. Based on this error, the network weights are updated via the intermediate layer from the output layer to the input layer (Singhal and Sharma, 2023).

#### 2.4.2 Genetic algorithm

The GA was employed (i) to optimize the MLP architecture design, including the optimal number of neurons, and (ii) to determine the optimal values of the input variables (B, BS, and WB ratios and running time) in the established MLP-GA models for the maximum RL and RR of shiitake.

**Figure 2:**
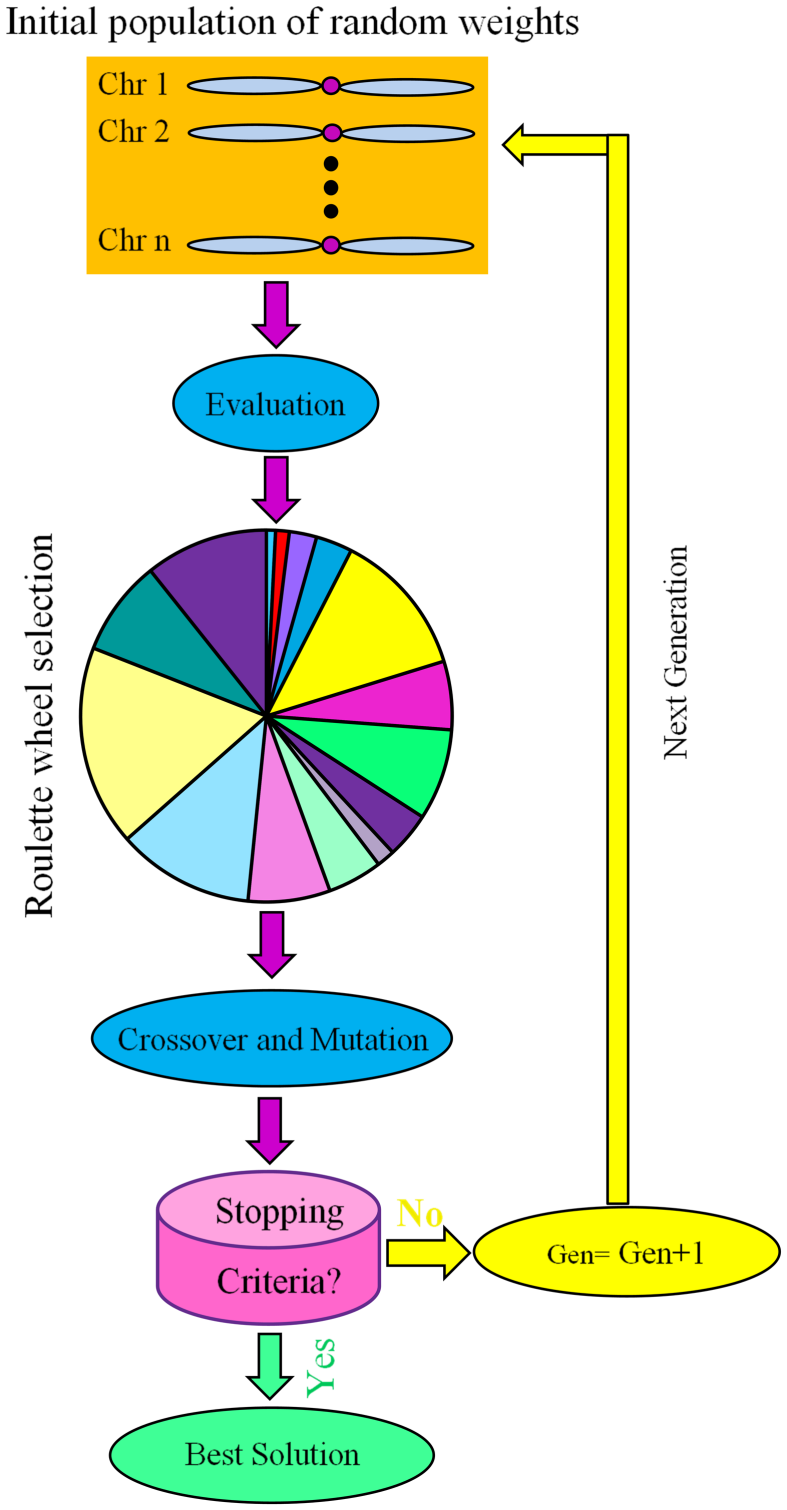
Schematic representation of genetic algorithm (GA) as an evolutionary optimization algorithm.

Holland (1992) proposed GA as a search strategy based on principles of natural selection (Goldberg, 1989; Sakawa, 2002; Guo et al., 2010). The GA begins with a population of random initial solutions (Figure 2). Each individual in a population is called a chromosome, which represents a possible solution to a problem. Chromosomes evolve over a series of iterations called generations. Chromosomes were assessed during each generation using fitness measurements (Figure 2). The next generation is created by crossing two chromosomes from the current generation or by changing one chromosome through a mutation, creating new chromosomes called offspring (Verma and Verma, 2012). To maintain a stable population size, a new generation is created by selecting parents and offspring based on fitness values and rejecting others. Fitter chromosomes have a higher chance of being selected. The best chromosome, which likely reflects the optimal solution to the problem, is obtained after several generations using algorithms (Guo et al., 2010).

The population size, crossover probability and mutation probability are the most important GA parameters. A generation number of 500, a crossover rate of 0.85, a mutation rate of 0.01, and an initial population of 50 (Haupt and Haupt, 2004; Abramson, 2007) were set to develop the fittest MLP structure and optimize the input variables (the ratios of B, BS, and WB, and running time) for the maximum output variables (the RL and RR of *L. edodes*).

**Figure 3:**
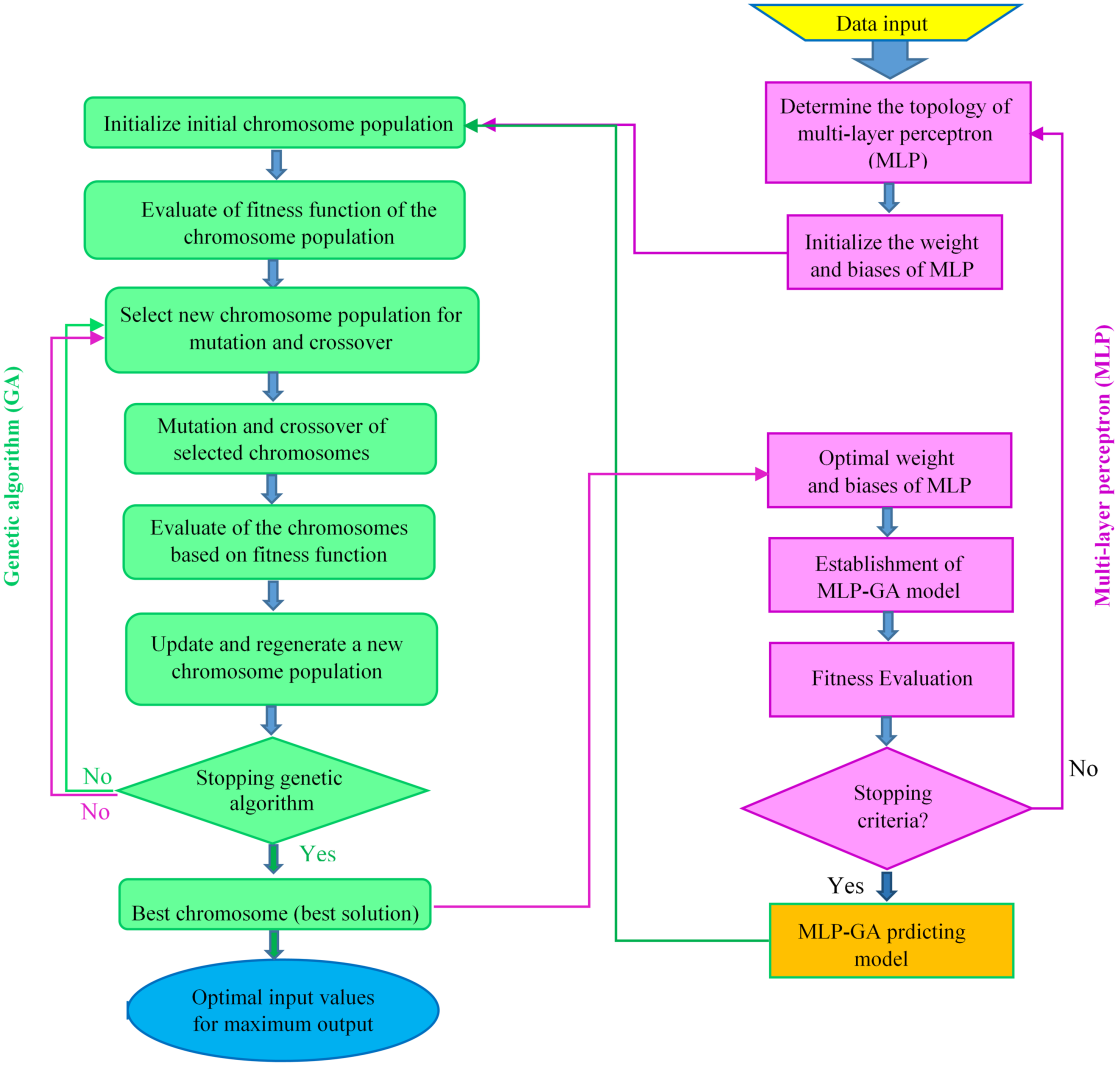
Flowchart of integrating multilayer perceptron (MLP) with genetic algorithm (GA) to optimize of the MLP architecture and input values to achieve the maximum level of each output.

The optimization strategy for finding the optimal MLP architecture and optimal values of input variables for the maximum RL and RR of *L. edodes* using the GA in the developed MLP-GA models is shown in Figure 3.

The performance of the MLP-GA models was evaluated using the coefficient of determination (R^2^), root mean square error (RMSE) and mean absolute error (MAE), as previously reported (Farhadi et al., 2020; Salehi et al., 2020a,b).

#### 2.4.3 Sensitivity analysis of the models

The MLP-GA models were subjected to sensitivity analysis to ascertain the degree of importance of the model input variables (ratios of B, BS, WB, and running time) on the model output variables, i.e., the RL and RR of *L. edodes*. The criterion used to determine the sensitivity of the ratios of B, BS, and WB and running time was the variable sensitivity error (VSE) value showing the performance (RMSE) of the MLP-GA model when a particular input variable was not available in the model. The variable sensitivity ratio (VSR) was derived as the ratio of the VSE and MLP-GA model errors (RMSE value) when all input variables were accessible. The estimated VSR values were then rescaled to fall within the range [0,1]. The highest importance variable of the model was the input variable with the highest VSR (Farhadi et al., 2020; Salehi et al., 2020a, 2021).

The MLP-GA and regression models were developed and evaluated mathematically using MATLAB (R2010a; MATLAB, 2010) and XLSTAT (XLSTAT, 2017), respectively, and graphics were created using GraphPad Prism 9 (2020).

### 2.5 Validation experiment

The ratios of bagasse, wheat bran, and beech sawdust and running time optimized by the GA were examined to assess the efficiency of the MLP-GA model in forecasting and optimizing the RL and RR of *L. edodes*.

## 3 Results

### 3.1 Effect of running time on *Lentinula edodes* running length

To determine the relationship between the running length (RL) and running time of *L. edodes*, the RL of shiitake cultured on growing substrate compositions (Table 1) was assessed at 5-d intervals after cultivation until the 40^th^ day. Overall, *L. edodes* RL increased during the running time and its highest significant level on S^25/50/0^, S^25/100/0^, S^25/100/25^, S^25/100/50^, S^25/100/100^, S^50/50/25^, S^50/50/50^, S^50/50/100^, S^50/100/0^, S^50/100/25^, S^100/100/25^, S^100/100/50^, and S^100/100/100^ was recorded on 25^th^ day after cultivation (Table 1). Accordingly, the highest statistically significant RL of *L. edodes* cultured on S^0/25/0^, S^0/25/25^, S^0/25/50^, S^0/25/100^, S^0/50/0^, S^0/50/25^, S^0/50/50^, S^0/50/100^, S^0/100/0^, S^0/100/25^, S^0/100/50^, S^0/100/100^, S^25/0/100^, S^25/25/0^ S^25/25/25^, S^25/25/50^, S^25/25/100^, S^25/50/25^, S^25/50/50^, S^25/50/100^, S^50/0/50^, S^50/0/100^, S^50/25/0^, S^50/25/25^, S^50/25/50^, S^50/25/100^, S^50/50/0^, S^50/100/50^, S^50/100/100^, S^100/25/0^, S^100/25/25^, S^100/25/50^, S^100/25/100^, S^100/50/25^, S^100/50/50^, S^100/50/100^, and S^100/100/0^ was measured 30 days after cultivation. Also, the highest significant RL of shiitake cultured on S^25/0/0^, S^25/0/25^, S^25/0/50^, S^50/0/0^, S^50/0/25^, and S^100/50/0^ was observed 35 days after cultivation. Besides, the highest statistically significant RL of *L. edodes* cultured on S^0/0/25^, S^0/0/50^, S^0/0/100^, S^100/0/0^, S^100/0/25^, S^100/0/50^, S^100/0/100^ was recorded on 40^th^ day after cultivation (Table 1).

### 3.2 Effects of different substrate compositions on *Lentinula edodes* running length

The effects of different growing substrate compositions on running length (RL) of *L. edodes* were explored in running-time- (*L. edodes* culture age), B-ratio-, WB-ratio-, and BS-ratio-dependent manners. As mentioned previously, the highest significant level of *L. edodes* RL was measured on 64 substrates. Accordingly, these time points were considered as a benchmark for the RL of *L. edodes* and were used in the subsequent statistical analysis. ANOVA showed that the main effects of the factors “B, WB and BS ratios” and their interactions (reciprocal and trilateral effects) on the RL (Supplementary Table S1) and RR (Supplementary Table S2) of *L. edodes* were highly significant. This suggests that the bagasse ratio affected the RL and RR of *L. edodes* differently at each ratio of wheat bran and beech sawdust or vice versa (i.e., the ratio of wheat bran and beech sawdust affected the RL and RR of *L. edodes* differently at each bagasse ratio). To examine these significant interaction effects, the B ratio was further analyzed on each ratio of WB and BS (Tables 2, 3).

**Table 2:** Effects of different ratios of growing substrate components on the running length (cm) of shiitake (*Lentinula edodes*).

Beach sawdust	Wheat bran (0%; 0 g)	Wheat bran (25%; 2.5 g)	Wheat bran (5%; 5 g)	Wheat bran (10%; 10 g)
**Bagasse (0%; 0 g)**				
0%;	0.00v	9.15 h-n	9.30 h-n	10.30abc
25%; 2.5 g	6.80r	10.10bcd	9.60e-j	9.75d-h
50%; 5 g	6.80r	9.62e-i	10.57a	9.90def
100%; 10 g	6.80r	9.02lmn	9.57e-k	10.60a
**Bagasse (25%; 2.5 g)**				
0%	5.20u	9.47f-m	9.77d-h	9.67e-i
25%; 2.5 g	5.20u	9.47f-m	10.45ab	9.57f-l
50%; 5 g	9.00lmn	9.72d-h	9.37 g-m	9.50f-l
100%; 10 g	9.07 k-n	10.02cde	9.15i-n	9.20i-n
**Bagasse (50%; 5 g)**				
0%	5.05u	9.60f-j	9.12j-n	9.82d-g
25%; 25 g	5.12u	9.60f-j	9.67d-i	9.75d-h
50%; 5 g	5.12u	9.72d-h	9.30 h-n	10.57a
100%; 10 g	7.20q	10.12bcd	9.27i-n	8.97mn
**Bagasse (100%; 10 g)**				
0%	5.97 t	9.45f-m	7.62p	9.35 g-m
25%; 2.5 g	6.05st	10.35abc	9.27 h-m	9.10 k-n
50%; 5 g	6.32 s	8.82no	9.25 h-m	9.62e-i
100%; 10 g	5.87 t	8.57o	9.25 h-m	9.60e-j

Means within a column followed by the same letter are not significantly different (*p* ≤ 0.05).

**Table 3:** Effects of the different ratios of growing substrate components on the running rate (cm d^−1^) of shiitake (*Lentinula edodes*).

Beach sawdust	Wheat bran (0%; 0 g)	Wheat bran (25%; 2.5 g)	Wheat bran (50%; 5 g)	Wheat bran (100%; 10 g)
**Bagasse (0%; 0 g)**				
0%;	0.000z	0.305n-r	0.310 L-q	0.343efg
25%; 2.5 g	0.170wx	0.337f-i	0.320j-o	0.325 h-l
50%; 5 g	0.170wx	0.321j-m	0.3525de	0.330 h-k
100%; 10 g	0.170wx	0.301pqr	0.319 k-o	0.353de
**Bagasse (25%; 2.5 g)**				
0%	0.150y	0.316 k-p	0.391a	0.387a
25%; 2.5 g	0.148y	0.316 k-p	0.348ef	0.383a
50%; 5 g	0.257 t	0.324i-m	0.312 L-q	0.380ab
100%; 10 g	0.302pqr	0.334 g-j	0.305n-r	0.368c
**Bagasse (50%; 5 g)**				
0%	0.144y	0.320j-o	0.304o-r	0.393a
25%; 25 g	0.146y	0.320j-o	0.387a	0.390a
50%; 5 g	0.171w	0.320j-o	0.372bc	0.352de
100%; 10 g	0.240u	0.338fgh	0.371bc	0.299qr
**Bagasse (100%; 10 g)**				
0%	0.149y	0.315 k-p	0.218v	0.312 L-q
25%; 2.5 g	0.151y	0.345efg	0.309 L-q	0.364 cd
50%; 5 g	0.158xy	0.294rs	0.308 m-r	0.385a
100%; 10 g	0.147y	0.286 s	0.308 m-r	0.384a

Means within a column followed by the same letter are not significantly different (*p* ≤ 0.05).

A mean comparison of the RL and RR of *L. edodes* cultured on the 64 substrates (Table 1) is presented in Tables 2, 3, respectively. Among the 64 substrate compositions used for *L. edodes* cultivation, the highest RL of *L. edodes* was recorded in substrates “S^0/100/100^, S^0/50/0^, S^50/100/50^, S^25/50/25^, S^100/25/25^, and S^0/100/0^” (Table 2). As shown in Table 3, S^25/50/0^, S^25/100/0^, S^25/100/25^, S^25/100/50^, S^50/50/25^, S^50/100/0^, S^50/100/25^, S^100/100/50^, and S^100/100/100^ exhibited the highest RR of *L. edodes* cultures on the different substrate compositions (Table 1).

### 3.3 Regression analysis

Mushroom growth is influenced by the type and ratio of substrate components (Ashrafuzzaman et al., 2009). Accordingly, optimizing the growing substrate composition is the main step to maximize the mycelial RL and RR of *L. edodes*. Predicting the optimal ratio of substrate components and running time is crucial for large-scale production of *L. edodes*.

**Figure 4:**
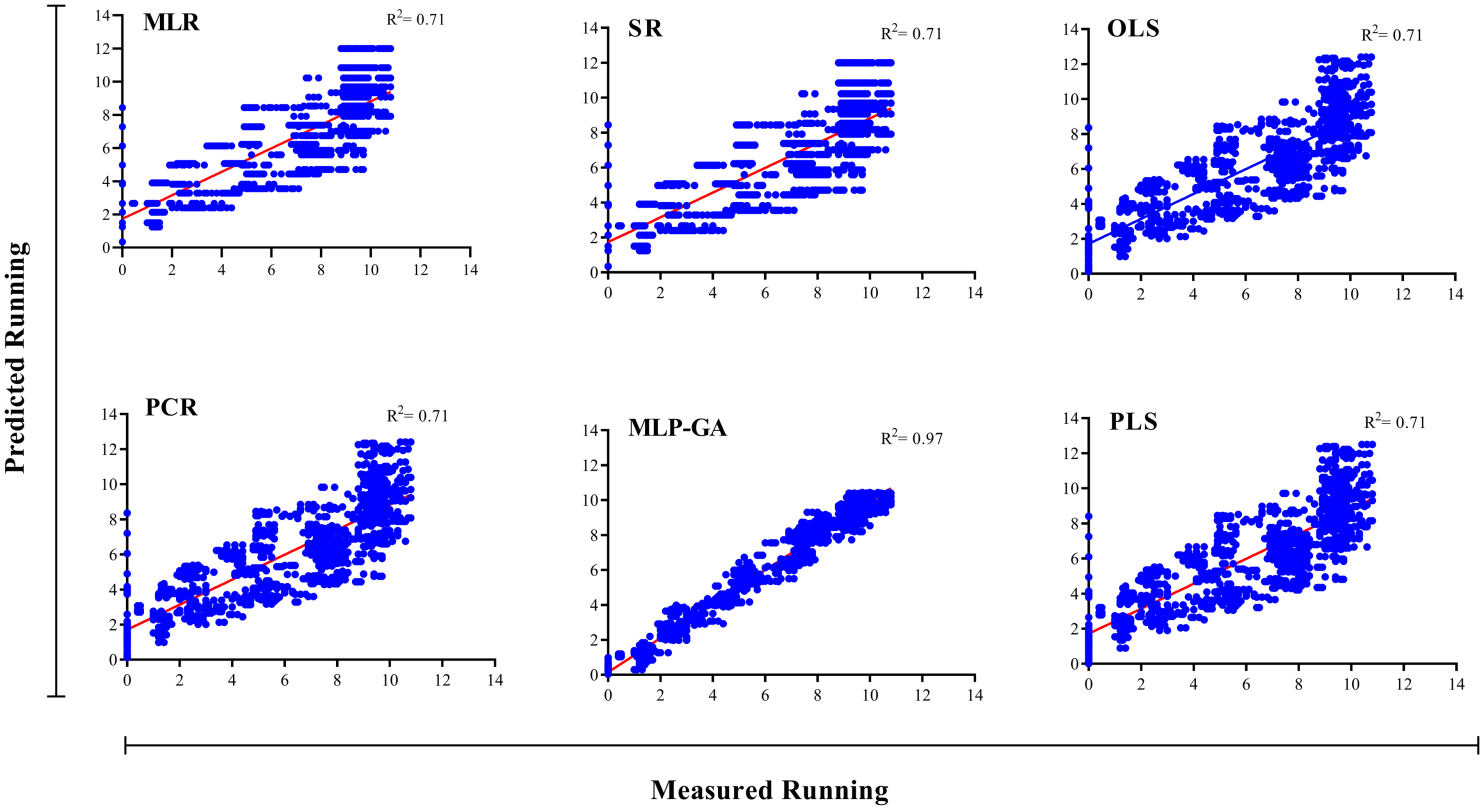
Scatter plot of actual data vs. predicted values of the running length of Shiitake (*Lentinula edodes*) using multiple linear regression (MLR), stepwise regression (SR), principal component regression (PCR), ordinary least squares regression (OLSR), partial least squares regression (PLSR), and multilayer perceptron-genetic algorithm (MLP-GA) models in the training subset. The solid line shows a fitted simple regression line for scatter points.

**Figure 5:**
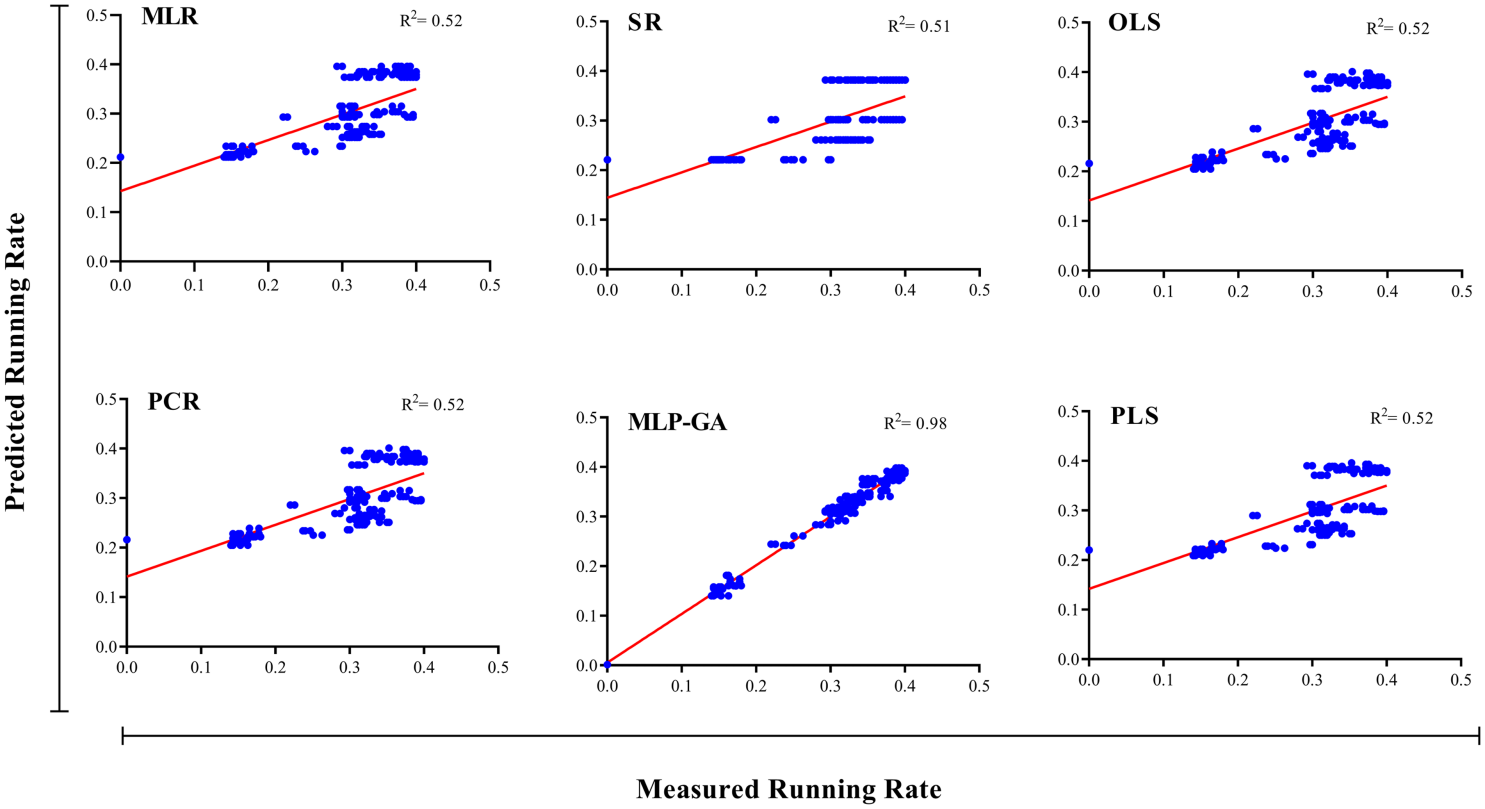
Scatter plot of actual data vs. predicted values of shiitake (*Lentinula edodes*) running rate using multiple linear regression (MLR), stepwise regression (SR), principal component regression (PCR), ordinary least squares regression (OLSR), partial least squares regression (PLSR), and multilayer perceptron-genetic algorithm (MLP-GA) models in the training subset. The solid line shows a fitted simple regression line for scatter points.

Different regression models (MLR, SR, OLSR, PCR and PLSR) were evaluated to determine the best regression method for predicting the RL and RR of *L. edodes* in response to the ratio of substrate components and the running time. All the regression models displayed statistically significant relationships between the input variables (ratios of B, BS, WB, and running time) and output variables “*L. edodes* RL and RR” (Table 4). The statistics of the developed MLR, SR, OLSR, PCR, and PLSR models for the RL and RR of *L. edodes* for the ratios of B, BS, and WB and running time are presented in Table 4. The goodness-of-fit, measured by R^2^, showed no difference in the predictive performance of the regression models developed for the RL and RR of *L. edodes* in the training and testing subsets (Table 4). The prediction accuracies of the MLR, SR, OLSR, PCR, and PLSR models were determined by plotting the actual (observed) values against the predicted values of the training subset (RL: R^2^ = 0.71, 0.70, 0.71, 0.71, and 0.71, respectively; RR: R^2^ = 0.52, 0.51, 0.52, 0.52, and 0.52, respectively; Figures 4, 5). No differences were observed in the prediction performance of the developed MLR, SR, OLSR, PCR, and PLSR models for the RL and RR of *L. edodes* (Figures 4, 5). The R^2^ values for the testing subset showed that the developed models accounted for 71% and 52% variability in RL and RR of *L. edodes*, respectively (Table 4).

**Table 4:** Statistics on multiple linear regression (MLR), stepwise regression (SR), principal component regression (PCR), ordinary least squares regression (OLSR), partial least squares regression (PLSR), and multilayer perceptron-genetic algorithm (MLP-GA) models for the running length and running rate of *Lentinula edodes* cultivated on different substrates obtained from different ratios of bagasse, wheat bran, and beech sawdust.

	Models	Training subsets			Testing subsets			Pr > F
		R^2^	RMSE	MAPE	R^2^	RMSE	MAPE	
Running length	MLR	0.71	1.88	1.53	0.71	1.88	1.54	<0.0001
	SR	0.70	1.87	1.53	0.71	1.88	1.54	<0.0001
	PLS	0.71	1.86	1.52	0.71	1.86	1.52	<0.0001
	PCR	0.71	1.87	1.52	0.71	1.86	1.52	<0.0001
	OLS	0.71	1.86	1.51	0.71	1.86	1.52	<0.0001
	MLP-GA	**0.97**	**0.54**	**0.43**	**0.97**	**0.60**	**0.47**	<0.0001
Running rate	MLR	0.52	0.06	0.05	0.53	0.05	0.04	<0.0001
	SR	0.51	0.06	0.05	0.51	0.05	0.04	<0.0001
	PLS	0.52	0.06	0.05	0.51	0.05	0.04	<0.0001
	PCR	0.52	0.06	0.05	0.53	0.05	0.04	<0.0001
	OLS	0.52	0.06	0.05	0.53	0.05	0.04	<0.0001
	MLP-GA	**0.98**	**0.01**	**0.01**	**0.92**	**0.02**	**0.01**	<0.0001

R^2^, coefficient of determination, RMSE, root mean square error, MAE, mean absolute error.

### 3.4 Multilayer perceptron-genetic algorithm analysis

First, the ratios of B, BS, and WB, and running time were used as input variables, and running length (RL) and running rate (RR) of *L. edodes* were used as output variables. The output variables were then predicted according to the established MLP-GA models. The performance of the developed MLP-GA models was evaluated by plotting the predicted values vs. the actual values for training (Figures 4, 5) and testing subsets (Table 4). Excellent agreement between the forecast and observed values of *L. edodes* RL and RR was observed for both the training and testing subsets (Figures 4, 5; Table 4).

The goodness-of-fit criterion results of the established MLP-GA models showed that the established models could accurately (R^2^ = 0.97, and 0.92) predict RL and RR of shiitake (*L. edodes*) in the testing subset, which was not utilized in the training processes of the MLP-GA models (Table 4). Furthermore, the established MLP-GA models exhibited balanced statistical values for the training and testing subsets (Table 4).

### 3.5 Sensitivity analysis of the models

VSRs were computed using all the data lines (training and testing subsets: 2,048 and 256 data points for the RL and RR, respectively) to list the input variables according to their relative importance in the model. VSRs were calculated for each of the output variables (*L. edodes* RL and RR) for the ratios of B, BS, and WB and running time (Table 5). Analysis of the RL model revealed that *L. edodes* RL was most sensitive to running time (VSR = 1.000), followed by WB ratio (VSR = 0.535), B ratio (VSR = 0.008), and WB ratio (VSR = 0.000). Accordingly, the RR of *L. edodes* was more sensitive to the WB ratio (VSR = 1.000), followed by the B ratio (VSR = 0.062) and the BS ratio (VSR = 0.000; Table 5).

**Table 5:** Importance (according to the sensitivity analysis) and optimal levels of the different factors, including bagasse ratio, wheat bran ratio, beech sawdust ratio, for achieving the maximum running length and running rate of *Lentinula edodes* cultured on different substrates using multilayer perceptron-genetics algorithm (MLP-GA) models.

	Variable (ratio)	Importance value (according to VSR^a^)	Optimal level	Optimal output
Running length	Bagasse	0.0081	15.11	10.69
	Wheat bran	0.5348	45.13	
	Beech sawdust	0.0000	10.16	
	Running time	1.0000	28.43	
Running rate	Bagasse	0.0628	30.68	0.437
	Wheat bran	1.0000	90.40	
	Beech sawdust	0.0000	0.00	

^a^Relative indication of the ratio between the variable sensitivity error and the error of the model when all the variables are available.

### 3.6 Mathematical optimization

The MLP-GA was linked to the GA to find the optimal levels of the input variables (ratios of B, BS, and WB and running time) for achieving the maximum RL and RR of *L. edodes*. The optimization analysis on the established MLP-GA models revealed that the substrate composition of 15.1% (1.51 g) bagasse, 45.1% (4.51 g) wheat bran and 10.16% (1.01 g) beech sawdust and the running time of 28 days and 10 h could result in the maximum *L. edodes* RL (10.69 cm; Table 5). The highest RR of *L. edodes* (0.44 cm d^−1^) could be obtained by a substrate containing 30.7% (3.07 g) bagasse, and 90.4% (9.04 g) wheat bran (Table 5).

### 3.7 Comparison of MLP-GA and regression models

The statistical values showed a higher prediction accuracy of the MLP-GA models compared with that of the regression models in terms of the calculated R^2^ for MLP-GA vs. regression models: RL = 0.97 vs. 0.71, and RR = 0.92 vs. 0.52 (Table 4).

### 3.8 Validation experiment

The RL of *L. edodes* cultured on S^15/45/10^ containing B (1.51 g), WB (4.51 g), and BS (1.01 g) for 29 d (optimized input variables in the MLP-GA model using the GA) was measured to be 10.81 cm ± 0.35 cm. Furthermore, *L. edodes* RR of 0.448 cm d^−1^ ± 0.039 was obtained on S^31/90/0^ containing B (3.07 g), WB (9.04 g), and BS (0.00 g).

## 4 Discussion

Forecasting the optimal ratio of substrate components is essential to enhance shiitake production and reduce costs. This is the first report to establish a mathematical model for the prediction of the RL and RR of *L. edodes* according to the ratios of growing substrate components (B, WB, and BS) and running time, as well as the optimization of growing substrate composition to achieve the maximum RL and RR. An accurate modeling system is required to forecast the optimal ratio of substrate components (B, BS, and WB) and running time for the maximum RL and RR of *L. edodes*. Various regression models and MLP-GA modeling were used to examine the relationships among the four input variables “ratios of B, WB, and BS and running time” and the output variables “RL and RR,” and the prediction probability of RL and RR using the studied input variables. Such mathematical forecasts have not yet been reported for valuable medicinal and edible mushrooms. Previous studies (Salehi et al., 2020a,b, 2021; Jahedi et al., 2023) have also indicated that MLP-GA models have superior accuracy compared with regression models (Figures 4, 5; Table 4). R-squared (Table 4), a measure of the fit of regression models for the testing subset, showed that these models could describe 71 and 52% variability in the RL and RR of shiitake, respectively, for unseen data. The results of the present study suggest that the developed MLP-GA models correctly predicted the RL and RR of *L. edodes* (R^2^ = 0.97 and 0.92, respectively; Table 4) in the testing subset, which was not utilized in the MLP-GA training process. The closeness of the errors of the training and testing subsets (Table 4), as well as the small number of hidden neurons confirmed the absence of overlearning during training and the good generalizability of the developed MLP-GA models for unseen data (Lou and Nakai, 2001; Farhadi et al., 2020; Salehi et al., 2020a, 2021). From the statistical metrics including R^2^ and RMSE of the training and testing subsets (Table 4), it can be inferred that the tansig activation function worked well for modeling throughout the experiment. Furthermore, the small RMSE values (Table 4) show the great potential of the developed MLP-GA models for predicting the parameters (output variables). Mycelium running is a colonization of the substrate thoroughly by fungal hyphae. The fungus first colonizes the entire substrate and then creates new hyphae by lateral branching, increasing the density and surface area of the colony. Fungi thrive in both nutrient-rich and nutrient-poor culture media. However, their lateral branching occurs only in the nutrient-rich medium, like what is observed in sparse and dense growth of fungal hyphae on water agar and potato dextrose agar. This could explain why the optimum substrate compositions for RL and RR are different.

Regardless of previous studies on the effects of growing substrate composition and running time on RL and RR of *L. edodes*, the question remains: which input variables are most important for RL and RR of *L. edodes*? As mentioned previously, sensitivity analysis showed that running time was the most important variable affecting mycelial RL of *L. edodes* (Table 5). The RR of *L. edodes* is important for commercial cultivation. Sensitivity analysis revealed that WB ratio was the most important factor affecting RR of *L. edodes* (Table 5). Shiitake running is a complex bioprocess that requires reliable methods for the model development and optimization. The MLP-GA has been successfully used to solve problems in various areas that are highly challenging and for which there is no known answer (Eftekhari et al., 2018; Salehi et al., 2020a; Jahedi et al., 2023). The growing interest in ANNs is primarily due to their ability to solve problems in various domains, their ability to model complex and nonlinear relationships, their ability to predict relationships on unseen data, and the fact that they do not require the specification of statistical data distribution (Mahanta, 2017). Due to the high prediction performance of the testing subset (Table 4), the established MLP-GA correctly predicted the RL and RR of *L. edodes*.

## 5 Conclusion

In this study, the RL and RR of *L. edodes* were modeled and optimized using mathematical methods for the first time. The high degree of fit between the forecasted and actual values of the output variables (the RL and RR of *L. edodes*) confirmed the superior performance of the developed MLP-GA models. This study presents MLP-GA as a useful mathematical tool for predicting and optimizing complex systems such as the growth of medical mushrooms, and the RL and RR of *L. edodes* in response to the composition of the growing substrate.

## Data availability statement

The original contributions presented in the study are included in the article/Supplementary material, further inquiries can be directed to the corresponding author.

## Author contributions

NS: Conceptualization, Investigation, Methodology, Project administration, Resources, Supervision, Writing – review & editing. MS: Conceptualization, Formal analysis, Investigation, Methodology, Validation, Visualization, Writing – original draft, Writing – review & editing. SF: Formal analysis, Investigation, Software, Visualization, Writing – review & editing. AA: Investigation, Methodology, Writing – review & editing. VM: Resources, Writing – review & editing.
