# Supplementary material for: Lentinula edodes substrate formulation using multilayer perceptron-genetic algorithm: a critical production checkpoint
Source: Front Microbiol. 2024 May 21;15:1366264. doi: 10.3389/fmicb.2024.1366264 (PMC11151849; doi:10.3389/fmicb.2024.1366264)
Supplement: Supplementary file 1 [file Data_Sheet_1.PDF]

Lentinula edodes Substrate Formulation using Multilayer Perceptron-Genetic Algorithm: A Critical Production Checkpoint

Naser Safaie[1*], Mina Salehi[2], Siamak Farhadi[3], Ali Aligholizadeh[1], Valiollah Mahdizadeh[1]

[1]Department of Plant Pathology, Faculty of Agriculture, Tarbiat Modares University, P.O. Box 14115-336, Tehran, Iran.

[2]Department of Plant Genetics and Breeding, Faculty of Agriculture, Tarbiat Modares University, Tehran, Iran.

[3]Seed and Plant Improvement Institute, Agricultural Research, Education and Extension Organization (AREEO), Karaj, Iran

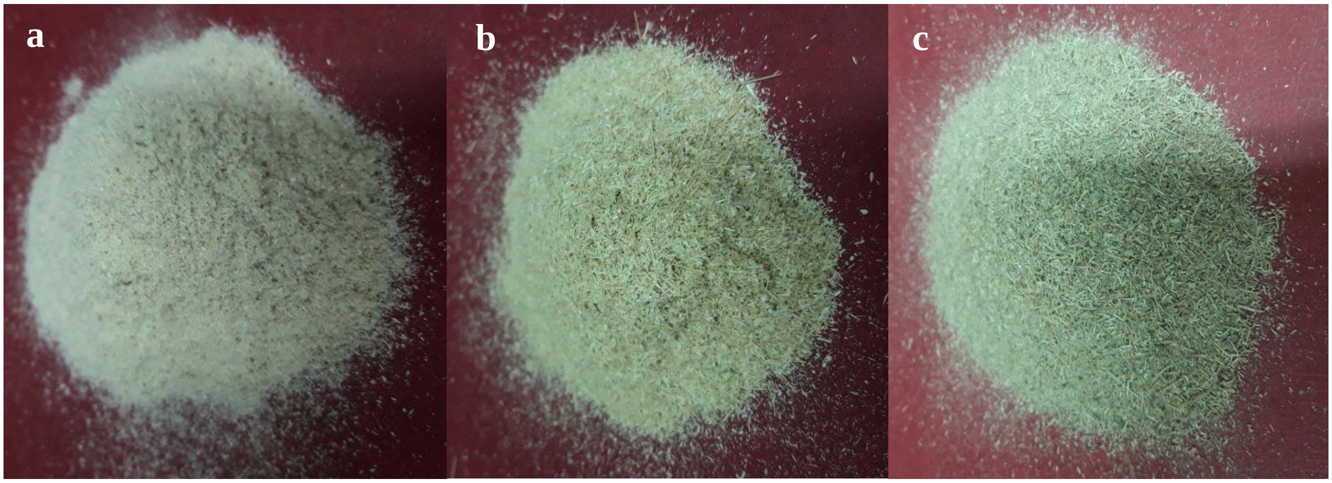

Fig S1. Powdered beech sawdust (a), wheat bran (b), and bagasse (c)

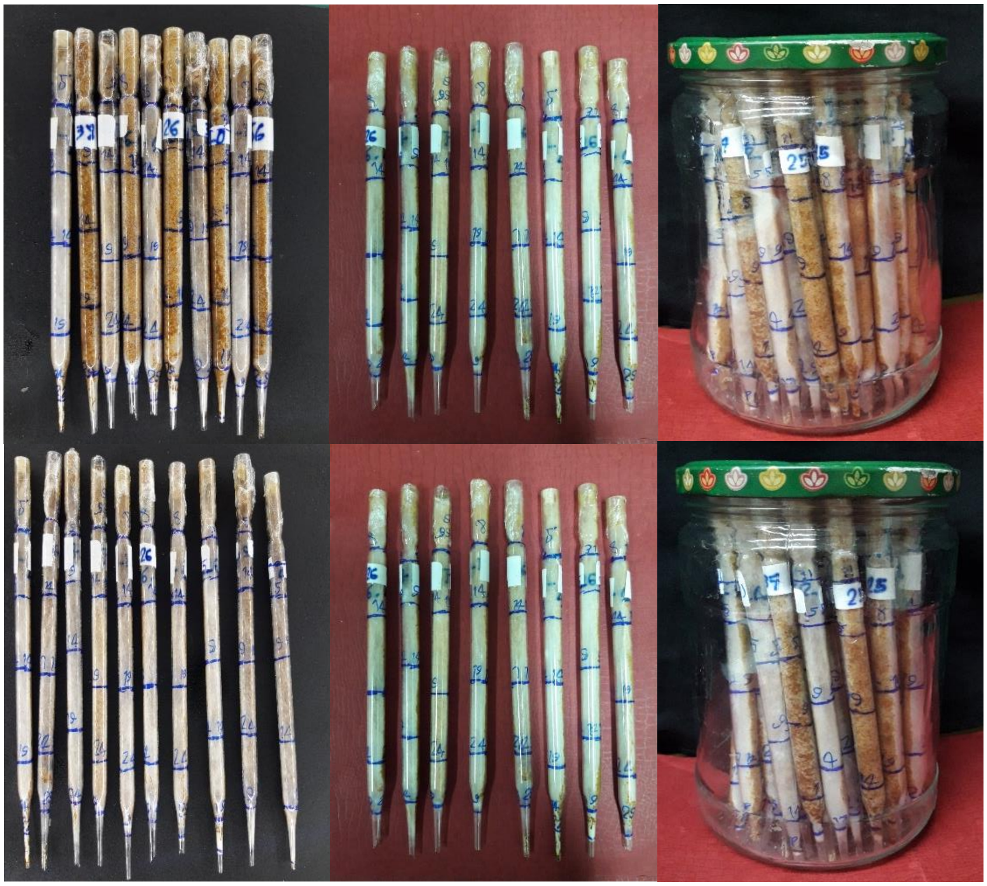

Fig S2. Shiitake (*Lentinus edodes*) cultured in Pasteur pipette containing the different substrates presented in Table S1

Table S1

Analysis of variance for the effects of different ratio of substrate components (bagasse, wheat bran, and beech sawdust) on running of shiitake (*Lentinula edodes*)

Source of variation	Degree of freedom	Mean square
Block	3	0.075^{ns}
Bagasse ratio (A)	3	3.842^{**}
Wheat bran ratio (B)	3	205.542^{**}
Beech sawdust ratio (C)	3	10.798^{**}
A × B	9	4.389^{**}
A × C	9	2.956^{**}
B × C	9	8.086^{**}
A × B × C	27	4.270^{**}
Error	189	0.040

*, ** and ns indicate significant difference p<0.05, significant difference p<0.01 and non-significant, respectively.

Table S2

Analysis of variance for the effects of different ratio of substrate components (bagasse, wheat bran, and beech sawdust) on running rate of shiitake (*Lentinula edodes*)

Source of variation	Degree of freedom	Mean square
Block	3	0.000111^{ns}
Bagasse ratio (A)	3	0.023035^{**}
Wheat bran ratio (B)	3	0.468968^{**}
Beech sawdust ratio (C)	3	0.012316^{**}
A × B	9	0.006459^{**}
A × C	9	0.002569^{**}
B × C	9	0.008533^{**}
A × B × C	27	0.006071^{**}
Error	189	0.000042

*, ** and ns indicate significant difference p<0.05, significant difference p<0.01 and non-significant, respectively.
